# Strain variation in feeding response of house flies, *Musca domestica*, to denatonium benzoate, a bittering agent used in commercial fly baits

**DOI:** 10.1371/journal.pone.0326572

**Published:** 2025-06-23

**Authors:** Panchalie B. Gunathunga, B. H. King, Edwin R. Burgess I.V.

**Affiliations:** 1 Department of Biological Sciences, Northern Illinois University, DeKalb, Illinois, United States of America; 2 Entomology and Nematology Department, University of Florida, Gainesville, Florida, United States of America; University of Leipzig Faculty of Life Sciences: Universitat Leipzig Fakultat fur Lebenswissenschaften, GERMANY

## Abstract

Granular fly baits remain one of the most popular and effective forms of chemical control of house flies (*Musca domestica*). While these baits contain a sucrose phagostimulant, some also contain the bittering agent denatonium benzoate at 20 or 100 ppm, as a feeding deterrent for humans. The response of adult house flies to 10, 100 and, 1000 ppm denatonium benzoate in sucrose solution was compared to response to sucrose only solution using proboscis extension response and no-choice consumption assays. Three house fly strains, DBQB, WD, and UF, each from a different Florida dairy farm, were tested within two generations of collection. Strain DBQB, but not strains WD or UF, had a known history of exposure to toxic baits containing denatonium benzoate, although recent insecticide history was unknown. For males, all strains avoided proboscis extension response and consumption with 1000 ppm, but not with 10 ppm. Males of WD and UF strains, but not DBQB strain showed significantly lower frequency of proboscis extension response at 100 ppm. Males of WD strain, but not DBQB or UF strains, also avoided consuming 100 ppm. For females, the pattern of significance for proboscis extension response was the same for all strains: avoidance for 1000 ppm but not for 10 or 100 ppm. Female consumption was significantly reduced at 1000 ppm for the DBQB and WD strains, but not for the UF strain, but no strain avoided 10 or 100 ppm for consumption. Thus, our results suggest that in some populations of house flies, 100 ppm of denatonium benzoate may reduce consumption by males, although not consumption by females.

## Introduction

House flies (*Musca domestica*) are pest insects that are a great nuisance to humans and livestock [1–3]. They occur in large numbers on farms in warm months [4] and in tropical areas hit by natural disasters [5]. They can transmit numerous pathogens, including *Salmonella* spp. and *Campylobacter* spp., to both humans and livestock [6–8]. Thus, controlling the population size of house flies is vital. Manure management, biological control methods, like use of parasitoid wasps, and chemical control measures, such as insecticidal fly baits, are used as pest management practices to control the population size of flies [3].

House fly baits, which are used as chemical control, include an active ingredient (insecticidal ingredient), a phagostimulant (feeding attractant like sucrose), sometimes a fly pheromone (e.g., (Z)-9-Tricosene) or another longer distance attractant, and sometimes a bittering agent [9–12]. As is the case with many pests, the evolution of resistance to chemical controls by house flies is a major concern. Much research on resistance is focused on the evolution of physiological resistance to the insecticidal ingredient. Physiological resistance mechanisms in pests include upregulation or mutations of detoxifying enzymes or structural alteration in insecticide binding sites [13,14]. Pests can also evolve behavioral resistance [15–17], which may involve avoidance of an insecticide formulation’s active ingredient [18–21]. Behavioral resistance may also evolve to inactive ingredients in insecticides. For example, populations of German cockroaches (*Blattella germanica* L.) evolved avoidance of glucose after having been exposed to glucose-containing baits for generations [22]. In these resistant cockroaches, response to glucose in sweet gustatory receptor neurons is greatly reduced, and glucose now activates neurons that normally respond to bitter compounds. Here we examine the feeding response to denatonium benzoate (DB) by adult house flies from three strains very recently established with field collected flies.

DB (CAS#3734-33-6; benzyl-[2-(2,6-dimethylanilino)-2-oxoethyl]-diethylazanium; benzoate), commercially known as Bitrex, is an inactive ingredient used in some house fly baits as a bittering agent [10,23]. DB is also used as an alcohol denaturant, as a bittering agent in household cleaners, pesticides, and in automobile products, as a safety measure to prevent accidental poisonings by humans (especially children) and pets. Making the product extremely bitter discourages swallowing of toxic substances [24–26]. DB is considered one of the most bitter compounds to humans: detectable at 10 ppb (= 0.01 ppm; 10 μg/L), judged slightly bitter at 50 ppb (= 0.05 ppm), and judged unpleasantly bitter at 10–20 ppm [26]. Exposure to DB at concentrations below 100 ppm has caused aversive responses in insects like vinegar flies (*Drosophila melanogaster*), German wasps (*Vespula germanica* Fabricius), codling moth caterpillars (*Cydia pomonella* L.), diamond back moth larvae (*Plutella xylostella* L.), and aphids (*Myzus persicae* Sulzer) [27–32]; whereas in commercial bait mixtures, DB is used at concentrations of 20 or 100 ppm, e.g., in QuickBayt 100 ppm is used and in Agita® GB fly bait 20 ppm is used [10,33].

Insects have taste receptors on various body parts. Adult house flies have taste receptors on their tarsi, not just their mouth parts [34–36]. House flies do not appear to taste with their antennae [37]. When the tarsi of a fly contact an appetitive food source, the tarsal taste receptors get stimulated, causing the fly to extend its sponging mouth parts, sometimes referred to as a proboscis extension response (PER) [38]. PER is often followed by consumption.

The present study examined PER and consumption of various concentrations of DB mixed in 10% sucrose solution, using three strains of adult house flies that had been recently collected from three different farms in Florida. The goal was to test, for each strain, whether males and whether females showed reduced feeding behaviors to each concentration of DB and then to consider whether the results seemed consistent with the usage history of house fly baits on the farm that the strain came from. One of the farms had a known history of using fly baits containing DB, whereas two farms did not. The response of adult house flies to bittering agents used in fly control products had previously only been studied in a single house fly strain that had been reared in the laboratory for years prior to testing [39]. This is the first study that looks into the response of adult house flies that are closer to “wild”.

## Methods

### Treatment solutions

Each PER experiment tested 0, 10, 100, and 1000 ppm of DB in sucrose solution, along with water. The consumption experiments used all but the last treatment, i.e., only 0 ppm of DB in sucrose solution was used as the control. A 10% (w/v) sucrose solution was used both for a control and as the diluent for DB because house flies readily feed on 10% sucrose solution [40].

A challenge in making the treatment solutions was completely dissolving the DB. Thus, first the required weight of DB (ThermoFisher Scientific, USA, catalog # J61048) (0.001 g for a 10 ppm solution, 0.01 g for a 100 ppm solution, 0.1 g for a 1000 ppm solution) was dissolved in 500 µL of methanol. Then all the methanol-DB mixture was added to 100 g of 10% sucrose solution. To avoid differential responses due to methanol, 500 µL of methanol was also added to the control of 0 ppm DB in 10% sucrose solution. The units for the treatment ppm are w/w, i.e., weight of DB per weight of the sucrose solution. The treatment solutions were placed under a fume hood for 24 h for methanol to evaporate. All treatment solutions were mixed on the same day.

### Collection and rearing of house flies

Three house fly strains were used in the experiments, each acquired from a different dairy in Northern Florida. The original collection of the house flies from the dairy farms did not require a permit and was done with permission from the dairy farms. Dairy West (WD) has used QuikStrike fly bait and has used PBO-synergized permethrin sprays once a week. WD also has a history of using Golden Malrin fly bait from July to October when fly numbers are high. The University of Florida calf unit (UF) has used QuikStrike fly abatement strips (nithiazine baits) during April to December for an unspecified number of years and PBO-synergized permethrin sprays year-round. Labels and Safety Data Sheets for QuikStrike fly bait and Golden Malrin fly bait do not mention containing DB as a bittering agent. Thus, the flies collected from WD and UF had no known history of exposure to DB. In contrast, the third strain (DBQB) was from a farm in Gilchrist County with a 2004–2007 history of intermittently using QuickBayt [10,23,41], which contains DB. But the insecticides that have been used at this farm in recent history are unknown.

Each fly strain used in the experiments was collected and then started as a laboratory colony in mid-May 2022. Adult house flies were collected using sweep nets. Adult house flies were reared in cages (47.5 x 47.5 cm BugDorms, MegaView Science, Taiwan) with sugar, milk powder, dehydrated egg and water *ad libitum*. The eggs were collected using 10 x 10 cm black cotton cloth placed over rearing media in adult cages. Eggs laid on the cloth were dislodged from the cloth with a stream of water into test tubes, from which 2 mL of eggs were transferred into larval media that consisted of a mixture of 355 g of Calf Manna (Manna Pro, Chesterfield, MO, USA), 1500 g of wheat bran, and 3750 mL of water [42]. Pupae that were 0–1 d old and from the F1 generation were shipped to the institution where experiments were done, and subsequent generations of each strain were reared there. The adult flies were reared in cages with dry sucrose, water, and diluted evaporated milk *ad libitum*. About 800 mm^3^ of eggs were transferred to a plastic box of wet larval media. The wet media was 2500 g of 13:1 w/w dry wheat bran:Calf Manna (Manna Pro, Chesterfield, MO, USA), along with roughly 1340 mL water. Pupae obtained were transferred to colony cages (nylon mesh cube: 30 cm^3^ from BugDorm, MegaView Science, Taiwan) for adult emergence. Upon emergence, and for the next 1–3 d, adult house flies that were to be used in experiments were provided with water *ad libitum* only. The adult flies used for experiments were first cold anesthetized by placing the cages in a freezer for about 2 min, until they fell to the bottom of the cage and stopped moving. Then they were transferred to a polystyrene Petri dish that was kept in crushed ice, where the flies were sorted into males and females using anatomical features of their external genitalia [43] and were fixed to wooden sticks for PER experiments or were placed in jars for consumption experiments. Each fly was tested only once and with only one treatment.

House fly response to DB was tested using PER and consumption measurements. For PER, flies from both F1 and F2 generations were used, whereas for consumption experiments flies from the F2 generation only were used.

### Proboscis Extension Response (PER)

Generally, a house fly’s first contact point with bait will be the tarsi. When the tarsal sensilla are stimulated by an appetitive substance, flies exhibit PER. Previous studies have shown that a higher percentage of flies exhibits PER when their tarsi contact sucrose versus water [38]. The PER experiments done in the present study tested, for each strain, whether the flies showed a significant decrease in PER when their tarsi contacted different concentrations of DB in 10% sucrose solution.

For PER experiments, the tip of a wooden stick (15.24 cm long, hardwood, Medichoice, USA) dipped in melted household paraffin wax (Gulf Wax, USA) was touched to the dorsal side of the thorax of an anesthetized fly. Flies recovered quickly as evident by their leg and wing movements [38]. Attaching flies using this method made it easier to move the flies to their treatments and to make sure that their tarsi were contacting the treatment.

The treatment solutions of 0, 10, 100, 1000, ppm of DB in 10% sucrose solutions, and water, were placed in concave wells (~18 mm diameter) [38] of glass slides, with enough solution surface area for all six tarsi of the fly to contact (approximately 20 µL). The observer was always blind to the concentration of the treatment. PER was recorded as occurring only when the fly extended its proboscis fully (as in S1 Fig [44,45]). Otherwise, “no PER” was recorded.

A randomized complete block design was used for each sex within each strain, where each block consisted of one replicate of each treatment, with the treatments tested one immediately after the other on the same day. The flies in each block were not only the same sex and strain, but also the same age and generation, and tested at the same temperature and relative humidity. Within each strain forty-one blocks were conducted for females, and forty-two for males. Testing was spread over the same ten different days for females of all strains and the same nine of those ten days for males of all strains. Thus, each day included flies of each strain and treatment, although the number of blocks tested on each day was not identical for each strain or sex, because of fly availability. Temperature and relative humidity were recorded. Each fly was only tested once and with just one of the treatment solutions.

### Consumption no-choice assay

This experiment examined consumption by flies from the three different strains to different concentrations of DB in the 10% sucrose solution. Treatment solutions tested were 0, 10, 100, 1000 ppm of DB. Consumption by flies was measured after their exposure to a treatment solution for 2 h. Specifically, the treatment solution was filled to the top of an amber glass vial (0.92 ml) [38]. Surface tension prevented spilling of solution in the vial due to movements of flies. Each vial was placed in a clear glass jar (237 mL). Then 10 house flies of the same sex were added to the jar, and the jar was covered with a white cloth held in place using an elastic band. Each block consisted of four treatment jars (1 jar for each concentration, all jars with flies) and four control jars (1 jar for each concentration, no flies). The control jars controlled for the effect of environmental conditions, like moisture absorption or evaporation, on weight measurements. No flies were added to these control jars. Treatment jars and control jars were kept inside an incubator maintained at 25 ± 0.05ºC with constant light for 2 h. The weight of each vial of solution was measured before and after the 2 h. Consumption was calculated for each concentration in each replicate as ((before weight of vial of solution in that concentration’s treatment jar – after weight of vial of solution of the same concentration) – (before weight of vial of solution in that concentration’s control jar – after weight of the vial of solution of the same concentration)).

After the 2 h of consumption, the number of dead flies was recorded. Thus, consumption per fly was calculated by dividing the consumption by the number of alive flies present in the jar at the end of 2 h. (However, if blocks with dead flies were discarded, conclusions were unaffected. Dead flies were found in only 5 of 120 jars, with only 1 or 2 flies dead in each of the 5 jars.) Blocks with spills were discarded.

A randomized complete block design was used for each sex within each strain. Each block consisted of one replicate of each treatment, with the treatments tested one immediately after the other on the same day and placed in the same part of the incubator at the same time. In each block, not only were the flies the same strain and sex, but also the same age and generation. Within each strain, tests were on at least three different days, the number of blocks was ten for each sex, and females and males were tested on the same days. Thus, each day included flies of each sex tested for each treatment, although because of fly availability, the number of blocks tested on each day was not identical for each sex and only two testing days were identical among strains.

### Statistical analyses

Statistical analyses were conducted in IBM SPSS Statistics for Windows, Version 25.0 [46]. We did only planned comparisons based on specific a priori hypotheses that the authors were interested in. Planned comparisons are “overwhelmingly recommended” over unplanned comparisons [47] to increase statistical power of each research question of interest without compromising on either Type I or Type II errors [48,49]. For each planned comparison significance was set at α = 0.05. In each experiment, for each strain, for each sex, response of house flies to each concentration of DB was compared to the sucrose only (0 ppm) control treatment as the research questions of interest were whether house flies of each strain show reduced feeding behaviors to denatonium-sucrose solutions compared to sucrose only control. We tested PER and consumption separately because it is already well documented from published literature that the response may be different for PER versus consumption [38]. We tested males and females separately because it is already well documented from published literature that they differ in what they PER to and consume [38]. For PER, each treatment solution was also compared to the water control. In PER experiments, comparisons were done using G tests, which are also called likelihood ratio tests. For consumption experiments, each comparison was with a paired t-test. Choice of paired t-tests was made before the experiment was done. Doing paired t-tests accounts for the blocked design of this experiment.

## Results

### Proboscis Extension Response (PER)

PER patterns of significance were similar for females of all three strains but not for males. Female house flies showed a significantly lower PER only at 1000 ppm of DB in 10% sucrose solution compared to 0 ppm (Table 1). Males of the DBQB strain also showed significantly less PER only at 1000 ppm of DB in 10% sucrose solution. But males of WD and UF strains both showed a significantly lower PER at 100 ppm, as well as at 1000 ppm, of DB in 10% sucrose solutions (Table 1).

**Table 1:** Percent of flies exhibiting Proboscis Extension Response (PER) in experiment with different ppm of DB (denatonium benzoate) in 10% sucrose solutions.

Treatment	Strain	Females						Males					
		PER	n	Compared with 0 ppm		Compared with water		PER	n	Compared with 0 ppm		Compared with water	
				G_1_	P	G_1_	P			G_1_	P	G_1_	P
**0 ppm**	WD	80%	41	–	–	38.45	**<0.001**	88%	42	–	–	42.96	**<0.001**
**10 ppm**	WD	68%	41	1.60	0.20	26.83	**<0.001**	81%	42	0.82	0.36	34.73	**<0.001**
**100 ppm**	WD	73%	41	0.62	0.43	31.16	**<0.001**	62%	42	7.68	**0.005**	18.02	**<0.001**
**1000 ppm**	WD	24%	41	25.87	**<0.001**	2.04	0.15	36%	42	24.43	**<0.001**	3.94	**0.045**
**water**	WD	12%	41	38.45	**<0.001**	–	–	17%	42	42.96	**<0.001**	–	–
**0 ppm**	DBQB	78%	41	–	**–**	35.90	**<0.001**	86%	42	–	**–**	22.01	**<0.001**
**10 ppm**	DBQB	71%	41	0.58	0.45	28.94	**<0.001**	83%	42	0.09	0.76	19.77	**<0.001**
**100 ppm**	DBQB	62%	42	2.57	0.11	21.91	**<0.001**	79%	42	0.73	0.39	15.76	**<0.001**
**1000 ppm**	DBQB	23%	40	24.99	**<0.001**	1.50	0.22	45%	42	15.22	**<0.001**	0.79	0.37
**water**	DBQB	12%	41	35.90	**<0.001**	–	–	36%	42	22.01	**<0.001**	–	–
**0 ppm**	UF	68%	41	–	**–**	26.83	**<0.001**	88%	42	–	**–**	35.21	**<0.001**
**10 ppm**	UF	78%	41	0.99	0.32	35.90	**<0.001**	76%	42	2.03	0.15	23.05	**<0.001**
**100 ppm**	UF	61%	41	0.48	0.49	21.03	**<0.001**	62%	42	7.68	**0.005**	12.44	**<0.001**
**1000 ppm**	UF	15%	41	24.32	**<0.001**	0.11	0.75	26%	42	32.86	**<0.001**	0.06	0.80
**water**	UF	12%	41	26.83	**<0.001**	–	–	24%	42	35.21	**<0.001**	–	–

Both females and males were 0–2-day old and tested at 19ºC - 24ºC, 33% − 42% RH.

df = 1 for all G tests.

In comparing PER to the various concentrations of DB in sucrose solution versus to water, the patterns of significance were mostly the same for all three strains and both sexes (Table 1). Specifically, a significantly greater proportion of individuals exhibited PER to the sucrose solutions with concentrations of 0, 10, 100 ppm of DB than to water. In contrast, sucrose solutions with 1000 ppm did not elicit PER in a significantly greater or lesser number of individuals than water did for any strain-sex, with the exception of WD strain males. WD strain males showed a significantly greater PER to sucrose solution even with 1000 ppm of DB compared to water.

### Consumption no-choice assay

**Fig 1:**
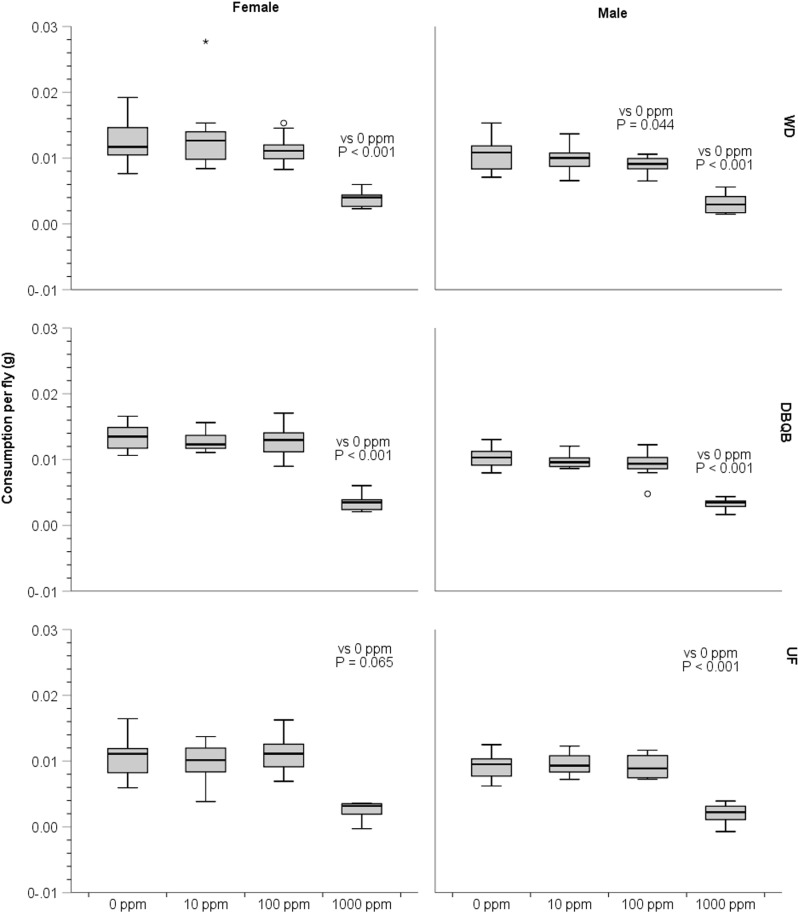
Consumption (g) of DB (denatonium benzoate) in 10% sucrose solutions compared to 10% sucrose only control. P values are from a paired t-test where each concentration was compared to 0 ppm control. P values are only shown for the paired t-tests that showed P < 0.05 or close to it (i.e., female UF 1000 ppm). n = 10 of each strain per sex. 1–2 – day old flies of F2 generation, 22ºC - 23ºC, 36% − 44% RH. In the box plot, the bottom line of the box represents the first quartile or the 25^th^ percentile, whereas the top line of the box represents the third quartile or the 75^th^ percentile. The middle line represents the median, and the distance between the top and bottom lines of the box is the interquartile range. The top horizontal line of the whisker represents the maximum value that is not an outlier, whereas the bottom horizontal line of the whisker represents the minimum value that is not an outlier. A circle represents a mild outlier, whereas a * represents an extreme outlier.

For the WD and DBQB strains, females showed a significantly lower consumption per fly at 1000 ppm of DB in 10% sucrose solution compared to the 0 ppm control (WD: t = 11.87, df = 9, P < 0.001; DBQB: t = 23.73, df = 9, P < 0.001) (Fig 1). Females of the UF strain did not show any significant difference in consumption per fly to any of the concentrations tested compared to the 0 ppm control, i.e., they readily consumed sucrose solutions regardless of the DB concentration (10 ppm: t = 1.16, df = 9, P = 0.277; 100 ppm: t = 0.96, df = 9, P = 0.36; 1000 ppm: t = 2.10, df = 9, P = 0.065). Males of the WD strain ate significantly less sucrose solution when it contained 100 ppm or 1000 ppm of DB (100 ppm: t = 2.35, df = 9, P = 0.04; 1000 ppm: t = 8.29, df = 9, P < 0.001), but not when it contained only 10 ppm (t = 0.84, df = 9, P = 0.42). In contrast, males of the DBQB and UF strains consumed significantly less sucrose solution only when the concentration of DB got as high as 1000 ppm (DBQB: t = 13.92, df = 9, P < 0.001; UF: t = 17.36, df = 9, P < 0.001) (Fig 1).

## Discussion

Despite the prior history of exposure to 100 ppm DB in baits, DBQB strain flies showed lower feeding response to only 1000 ppm of DB. In contrast, the two strains that appear to have lacked such exposure, WD and UF, showed lowered feeding response to 100 ppm (WD and UF males in PER, WD males in consumption) and 1000 ppm of DB (except for UF females in consumption). However, it is important to note that what baits were used on the DBQB farm recently, in the ~ 10 years before our collection, is unknown.

The differences seen among the strains tested in the present study suggest it may be worth looking further into the evolution of taste avoidance to bittering agents by house flies. The DBQB strain was exposed to baits containing DB ~ 10 + years before we tested them [41], whereas WD and UF strains were not exposed to granular baits containing DB in recent history. However, we cannot rule out the possibility that there are other historical differences among the strains as well, e.g., in exposure to other bitter compounds. Thus, it would be worthwhile to more directly investigate whether fly populations evolve altered taste perception of DB after generation(s) of exposure to an insecticidal bait containing this bittering agent.

For all three strains, WD, DBQB, and UF, 1000 ppm of DB in 10% sucrose solution was significantly less appetitive relative to 0 ppm DB, and this was true for males and females based on PER (Table 1) and consumption (except for UF females) (Fig 1). Nevertheless, a small percentage of flies exhibited PER even to this high concentration.

Sex differences in taste are documented for dipterans [50,51]. We did not directly compare males and females. However, based on our results, we hypothesize that males are generally more sensitive than females to DB. For WD and UF males, DB appears to reduce appetitiveness of 10% sucrose solutions at lower concentrations compared to females. In contrast, the DBQB strain, which has a history of exposure to baits containing DB, showed no significant difference in PER at 100 ppm DB to the sucrose-only control, for both males and females. In the consumption experiments, at 100 ppm of DB in 10% sucrose solution, males of WD strain showed a significantly lower consumption compared to sucrose only control, whereas females did not (Fig 1). In contrast to the WD strain, both females and males of UF and DBQB strains showed no significant difference in consumption at 100 ppm of DB compared to the sucrose-only control. Thus, DB in commercial toxic house fly baits seems more likely to reduce feeding by male than female house flies in at least some populations. If males, not females, are more sensitive to DB, this may be fortunate in terms of the efficacy of house fly baits that contain DB. Bait formulation efforts may especially target females, as females pose the greatest risk of increasing population size, at least in the many species where females only have to mate once, and a single male can inseminate many females. Female house flies also carry more bacterial pathogens compared to male house flies, thereby posing a greater risk than males as vectors for human and animal pathogens [52,53]. The apparent sex difference in feeding response to DB of house flies in the present study cannot be easily explained by what is currently known about house fly diet or mating [54,55]. In *D. melanogaster* sexual dimorphism related to trehalose sensitivity has been linked to the *Tre* gene [51], and males have more taste sensilla on their legs than do females [56]; but these have not yet been examined in house flies.

PER to (but not consumption of) different bitter-sucrose concentrations was also compared to response to water. All strains and both sexes seemed more interested in sucrose than in plain water, probably because they had been food-deprived but not water-deprived prior to testing. Consistent with this, the house flies exhibited detectably higher PER to 10% sucrose solutions than to water when the sucrose solution had one of the three lower concentrations of DB (0, 10 and 100 ppm) (Table 1). There may be a threshold beyond which DB interferes with the appeal of sucrose, and the order of magnitude of that threshold may be about 1000 ppm or higher.

Given the results of the present study, the concentrations 100 ppm and 1000 ppm of DB may not be good concentrations to be used in fly bait mixtures as bittering agents, as flies tended to avoid those concentrations relative to plain sucrose. On the other hand, given that current insecticidal house fly baits seem to contain 20 or 100 ppm [10,33], it is encouraging that 61–79% of the flies showed PER to 100 ppm of DB in sucrose solution. The PER response (the initial step in fly feeding behavior) can be increased by decreasing the concentration of DB. Humans find DB unpleasantly bitter at a concentration of 10–20 ppm [26]. Thus, a concentration less than 100 ppm and greater than 20 ppm should be tested with house flies from a variety of farms to see what concentration within this range flies do not avoid in a bait mixture. The bittering agent DB is marketed as a repellent for children. Additional study of pet responses is needed. Dogs appear to be less sensitive to DB than humans but still avoid 47.5 ppm of DB in a water choice test [57]. What concentration is avoided when DB is mixed with sucrose remains to be seen.

At farms using baits with DB, avoiding DB, at least at concentrations like those used in baits, may be adaptive for house fly populations. However, avoiding lower concentrations may also be adaptive for house flies at such farms. When bait is exposed to moisture, its components may disseminate, and the concentration of those ingredients may be lower at greater dissemination distances. This has been shown for imidacloprid in QuickBayt [58]. Whether the DB does the same while staying associated with the formulated bait, thus potentially killing any flies that do not avoid the associated bitterness, remains to be seen. In the present study we found that males from some field populations of house flies avoid DB at concentrations found in often-used toxic bait. Further research is recommended to see whether the efficacy of the entire commercially available bait with DB, not just a sucrose and DB mixture, is low on farms with a clear recent history of DB containing baits. In a strain of laboratory reared house flies, response to DB varies based on the diluent used, with reduced PER to 100 and 1000 ppm of DB when dissolved in sucrose, but only to 1000 ppm of DB when dissolved in orange juice [39]. Thus, house fly response to currently used concentrations of DB might differ when in bait formulae versus in sugar solution. Future research on house fly response to different concentrations of DB in bait formulae is recommended. A currently common recommendation for controlling house flies and other pests is to rotate the toxin (killing agent) used, to prevent the evolution of resistance. Perhaps rotation of other components of insecticide formulations, including the bittering agent DB, might also be beneficial. A promising alternative bittering agent is sucrose octaacetate [39].

## Supporting information

S1 Fig *Musca domestica* on a stick with tarsi contacting solution and with proboscis fully extended. Picture by Elizabeth Taylor, Northern Illinois University.
(PDF)

S1 Table PER by house flies FL data sheet. (XLSX)

S2 Table Consumption by house flies FL data sheet. (XLSX)
