# Supplementary material for: Strain variation in feeding response of house flies, Musca domestica, to denatonium benzoate, a bittering agent used in commercial fly baits
Source: PLoS One. 2025 Jun 23;20(6):e0326572. doi: 10.1371/journal.pone.0326572 (PMC12184930; doi:10.1371/journal.pone.0326572)
Supplement: S1 Fig — Picture by Elizabeth Taylor, Northern Illinois University. (PDF) [file pone.0326572.s001.pdf]

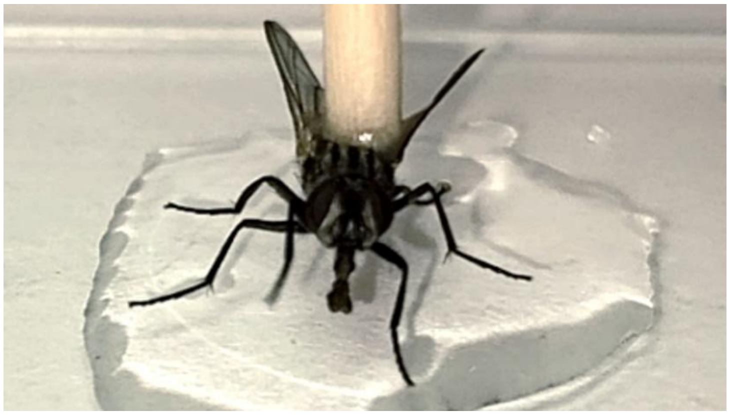

S1 Fig. *Musca domestica* on a stick with tarsi contacting solution and with proboscis fully extended.
